# The Associations of Serum Osteocalcin and Cortisol Levels With the Psychological Performance in Primary Hyperparathyroidism Patients

**DOI:** 10.3389/fendo.2021.692722

**Published:** 2021-08-12

**Authors:** Shu-min Wang, Yang He, Min-ting Zhu, Bei Tao, Hong-yan Zhao, Li-hao Sun, Jian-min Liu

**Affiliations:** Department of Endocrine and Metabolic Diseases, Rui-jin Hospital, Shanghai Institute of Endocrine and Metabolic Diseases, Shanghai Clinical Center for Endocrine and Metabolic Diseases, Shanghai Jiao Tong University School of Medicine, Shanghai, China

**Keywords:** depression, anxiety, primary hyperparathyroidism, cortisol, osteocalcin

## Abstract

**Objectives:**

The aim of this study was to investigate factors responsible for the psychological performance in primary hyperparathyroidism (PHPT) patients.

**Methods:**

A group of 38 PHPT patients receiving questionnaires, including Beck Depression Inventory (BDI), State–Trait Anxiety Inventory (STAI), and 36-Item Short Form Survey (SF-36), was evaluated. The relationships between scores of questionnaires and clinical biomarkers were examined. Collinearity and linear regression model were applied to examine variables determining the scores of the questionnaire. In 192 PHPT patients, bivariate and partial correlation were used to analyze the relationships between serum concentrations of parathyroid hormone (PTH), calcium, osteocalcin (OCN), and cortisol.

**Results:**

Among 38 patients receiving questionnaire tests, 50% (19/38) of the patients developed state anxiety, 60.5% (23/38) of the patients had the trait of developing anxiety. In addition, 18.4% (7/38) of the patients developed mild to severe depression. Serum cortisol at 8:00 was negatively and significantly correlated with social function (r = -0.389, p = 0.041) after controlling for age, sex, disease duration, serum PTH, calcium, phosphorus, and 25-hydroxyvitamin D [25(OH)D] concentration. OCN was significantly and negatively correlated with score of STAI-S (r = -0.426, p = 0.027). In the linear regression model for BDI score, variables with statistical significance were serum OCN (β = -0.422, p = 0.019) and cortisol at 0:00 (β = 0.371, p = 0.037). In 192 PHPT patients, the serum concentration of OCN (r = 0.373, p = 0.000) was positively correlated with PTH level. After controlling for age, sex, disease duration, serum 25(OH)D, phosphorus, and calcium concentration, the positive correlation between OCN and PTH was still statistically significant (r = 0.323, p = 0.000). The serum concentration of cortisol at 0:00 was significantly and positively correlated with serum calcium (r = 0.246, p = 0.001) in bivariate correlation analysis. After controlling for age, sex, disease duration, serum PTH, 25(OH)D, and phosphorus concentration, serum cortisol at 0:00 was still positively and significantly correlated with serum calcium (r = 0.245, p = 0.001).

**Conclusion:**

Serum levels of OCN and cortisol, rather than PTH and calcium, are associated with the development of anxiety and depression symptoms in PHPT patients.

## Introduction

Primary hyperparathyroidism (PHPT) is a disorder of parathyroid hormone (PTH) hypersecretion by parathyroid gland(s) in patients with normal renal function, resulting in increased serum calcium concentration (1). While most patients today are “asymptomatic”, lacking the classical skeletal and renal manifestations of PHPT, nonspecific neuropsychological symptoms are also investigated (2–5). Several studies suggested that PHPT was associated with impaired quality of life (QoL), anxiety, and depression as evaluated by questionnaires (6–8). Although these symptoms are concerning, there is a debate upon whether these symptoms are directly and specifically attributable to PHPT (7). There were mainly two facts contributing to this argument. For one thing, there is no consistent difference in the psychological performance between PHPT patients and control counterparts (7–9). For the other, the reversible role of parathyroidectomy on these psychologic features was not fully recognized (1, 9–11). Therefore, at the Third International Workshop on Asymptomatic Primary Hyperparathyroidism (12), studies on the psychological and cognitive features of PHPT were reviewed and were not considered to be an indication for parathyroidectomy (13). As for the primary exploration for factors of PHPT neuropsychological manifestations, studies were mainly concentrated on the relationship between psychological performance and PTH or calcium. However, neither the increased PTH nor calcium concentration was definitely reported to be the direct cause (1, 7).

In fact, a number of hypotheses have been proposed with regard to the mechanism of depression and anxiety development. “Hypothalamus pituitary adrenal (HPA) axis disorder” hypothesis (14) was a classic and widely accepted hypothesis for the pathophysiology of anxiety and depression. Glucocorticoids exerted damaging effects on psychological function. Mouse experiments showed that high-dose glucocorticoid reduced the neurogenesis of the hippocampus and olfactory bulb that were related to depression and anxiety behaviors (15–17). For humans, Cushing’s syndrome (CS) was a pathological model of hypercortisolemia. The psychiatric feature of hypercortisolism is a well-recognized manifestation of CS, as described decades ago (18). In a study by Kelly et al. (19), including 209 patients with active CS of all ages, depression was present in 57% of the patients, while anxiety was diagnosed in 12% of the patients. In addition to cortisol, recently, the beneficial effects of osteocalcin (OCN), a bone-derived protein, on improving neurological performance were reported, such as cognition impairment (20, 21), neuromotor dysfunction (22), and anxiety and depression (21, 23). However, the changes of cortisol and OCN concentration in PHPT, especially their relationships with psychological features in PHPT patients, have not been investigated.

In this study, both serum cortisol and OCN concentration changes and the correlation of psychological features with serum OCN and cortisol were analyzed in a group of PHPT patients.

## Materials and Methods

### Patients

In this study, psychological questionnaires, which were not a mandatory requirement for every patient in our department, were tested from August 2020. Until December 2020, questionnaire information in 38 out of 52 patients in this period was obtained. The inclusion criteria included: i) elevated serum calcium level with inappropriately high serum PTH level and ii) with a complete record of serum PTH, OCN, and cortisol concentration. The exclusion criteria included: i) secondary and tertiary hyperparathyroidism, ii) multiple endocrine neoplasms, iii) malignancy, iv) chronic kidney disease (CKD) stages 4 and 5 or eGFR ≤30 ml/min, v) a history of head trauma or stroke, and vi) medications with glucocorticoid. In order to find out whether serum concentration of OCN and cortisol changed as PTH and calcium increased in PHPT patients, a total of 192 PHPT patients admitted to our department from January 2011 to December 2020, including the above 38 patients, were retrospectively evaluated, who met the above inclusion and none of the exclusion criteria. All the patients were managed by the standard protocol developed by our department. Due to the retrospective nature of this study, the written informed consent was waived, which was approved by the ethics committee of our hospital (2017-201).

### Clinical Features

The age of onset was recorded according to the first identification of symptoms related to PHPT (bone pain, nephrolithiasis, pathological fractures, polydipsia and polyuria, digestive symptoms, neuropsychiatric manifestations, etc.) or an elevation in serum calcium or PTH concentrations. The anthropometric information was also collected, including sex, age at the time of diagnosis, and body mass index (BMI).

### Biochemical Markers and Bone Mineral Density Measurements

Blood samples were collected in the morning after 10 h of fasting. Fasting serum albumin, calcium, phosphorus, and creatinine levels were measured using an automatic biochemical analyzer (Beckman Coulter, DXH 800, USA). Serum level of PTH was measured by intact immunoradiometric assay (ARCHITECT i2000sr, Abbott, Chicago, IL). Serum level of 25-hydroxyvitamin D [25(OH)D] was measured by electrochemiluminescence immunoassay (Roche Diagnostics, Indianapolis, IN, USA). Serum level of OCN was assayed by two methods: radioimmunoassay (Gamma radioimmunoassay counter GC-911, ZONKIA, China) and electrochemiluminescence (Cobas, E601, Roche) during different periods. Serum concentrations of Type I procollagen amino-terminal peptide (PINP) and Collagen I telopeptide-β (β-CTX) were examined through electrochemiluminescence method (Cobas, E601, Roche). Serum concentrations of cortisol (collected at 8:00, 16:00, and 0:00) and 24-h urinary cortisol were assayed by chemical luminescence assay (Beckman Coulter Corp., Brea, CA, USA). Plasma adrenocorticotropic hormone (ACTH) level was tested by chemical luminescence assay (Mindray CL-600i, China). Area bone mineral densities (aBMDs) at the lumbar spines 1–4 (L1–L4) were measured by dual-energy X-ray absorptiometer (DXA, Lunar Prodigy; GE Medical Systems).

### Psychological Testing in Primary Hyperparathyroidism Patients

Testing was conducted preoperatively by a doctor who was not blinded to disease state, lasting approximately 0.5 h for each. Psychological tests included State–Trait Anxiety Inventory (STAI) (24), Beck Depression Inventory (BDI) (25), and 36-Item Short Form Survey (SF-36) (9). In our study, only mental components (MCs) of SF-36 were analyzed, including social function (SF), role of emotion (RE), mental health (MH), and vitality (VI).

The STAI (24) measures anxiety and consists of two 20-item scales measuring trait anxiety (anxiety proneness) and state anxiety (a current emotional condition), with higher scores suggesting more obvious anxiety or more traits developing anxiety. As a reference, mean raw values ( ± SD) for working adults aged 50–69 years are 32.2 ± 8.7 for state anxiety and 31.8 ± 7.8 for trait anxiety. For BDI (25), higher scores indicate more symptoms: 0–13 indicates no or minimal depression; 14–19, mild depression; 20–28, moderate depression; and 29–63, severe depression. For items in SF-36 (9), a higher score indicates a better QoL; for mental components of SF-36, a higher score suggests better psychological performance.

### Statistical Analysis

For results in this study, continuous variables were expressed as means ± SD or median (minimum, maximum) according to their distributions. Categorical variables were summarized as group number/total number. The comparisons of continuous variables between groups were performed using t-test or one-way ANOVA for normally distributed variables; otherwise, nonparametric test. Categorical data were compared by chi-square test. As serum concentration of OCN was tested by two different kinds of methods, OCN was transformed into categorical variables according to its tertile values. Pearson correlation analysis (two-tailed) was used to investigate the relationship of PTH, calcium with cortisol, as well as the association between the serum PTH, calcium, OCN, cortisol levels, and parameters of questionnaires. Kendall’s tau-b was used to test the correlation between OCN and PTH or calcium, which were also transformed into categorical variables here. Partial correlation analysis (two-tailed) was used to examine the above associations when controlling for clinical features and biomedical markers. Linear regression analysis with backward mode was applied to examine determining variables of questionnaire score. In this part, age; sex; disease duration; serum PTH; calcium; phosphorus; 25(OH)D; OCN; cortisol of 8:00, 16:00, 0:00; and 24-h urinary free cortisol were considered. To get stable results, collinearity analysis was used to eliminate collinear variables in each questionnaire regression model. The cutoff value of the variance decomposition proportion for the diagnosis of multicollinearity is set to 0.3 in dimensions with condition index over 10 according to the work of Liao (26) and Kim (27). After screening, all the variance inflation factors became less than 2, indicating that no multicollinearity existed (26, 27). All statistical calculations were performed using the SPSS (version 23.0; IBM statistics). A p-value <0.05 was considered statistically significant.

## Results

### Higher Serum Cortisol Concentration and Lower Serum Osteocalcin Level Were Correlated With Worse Psychological Manifestation in Primary Hyperparathyroidism Patients

Since only 38 out of 52 patients received psychologic evaluation, including BDI, STAI, and SF-36 questionnaire during August 2020 to December 2020, a sensitivity analysis was performed between patients who had taken the tests and those who had not. As shown in the **Supplementary Table**, there were no between-group differences in age, sex, disease duration, body weight, height, BMI, systolic blood pressure (SBP), heart rate, BMD (L1~L4), serum concentrations of PTH, 25(OH)D, β-CTX, albumin, hemoglobin, HbA1c, and phosphorus (all p > 0.05). Serum calcium concentration (p = 0.006) was higher in patients receiving questionnaires. However, serum and urine cortisol concentrations had no statistical difference between the two groups (p > 0.05).

For PHPT patients receiving questionnaire tests, the SF-36 MC items’ scores were 74.04 ± 23.32 for SF, 72.74 ± 18.71 for MH, 57.76 ± 22.17 for VI, and 66.67 (0,100) for RE, respectively. The score of STAI-S was 35.43 ± 11.56, STAI-T 37.39 ± 10.34, and BDI was 5 (0,46). According to the reference (24), out of the 38 patients receiving questionnaires, 50% of the patients (19/38) with a STAI-S score over 32.2 were in a state of anxiety; 60.5% of the patients (23/38) with the score of STAI-T over 31.8 have the trait of developing anxiety; 44.74% of the patients (17/38) had both STAI-S over 32.2 and STAI-T over 31.8. As for BDI score (25), 18.4% patients (7/38) developed mild to severe depression. Here, 15.9% of the patients (6/38) developed both anxiety (STAI-S and STAI-T were higher than the reference) and depression.

In addition, we assessed associations of PTH, calcium, OCN, and cortisol concentrations with psychological performance, as evaluated by questionnaires. As shown in **Table 1**, in bivariate model, cortisol concentration at 8:00 was significantly and negatively correlated with SF (r = -0.393, p = 0.015), one feature of psychological health evaluation. Serum OCN concentration was significantly and negatively correlated with BDI (r = -0.345, p = 0.0378). Serum concentration of PTH and calcium was not correlated with any of the scores in the questionnaires (p > 0.05). After controlling for age, sex, and disease duration, serum concentration of cortisol at 8:00 was still significantly and negatively correlated with SF (r = -0.391, p = 0.020). When serum PTH, 25(OH)D, phosphorus, and calcium were further adjusted, the correlation of cortisol at 8:00 with the score of SF (r = -0.389, p = 0.041) was still significantly negative; the negative correlation of serum OCN concentration and STAI-S (r = -0.426, p = 0.027) was statistically significant.

**Table 1:** Correlation between scores of questionnaires and biomarkers in PHPT patients.

	SF		RE		MH		VI		STAI-S		STI-T		BDI	
	r	p	r	p	r	p	r	p	r	p	r	p	r	p
Model1														
PTH	0.071	0.0.674	-0.175	0.0.293	0.172	0.0.303	0.156	0.0.349	-0.148	0.0.383	-0.168	0.0.328	-0.166	0.0.327
Calcium	-0.120	0.480	0.066	0.698	0.033	0.844	0.063	0.710	-0.060	0.726	0.049	0.782	0.027	0.874
Cortisol at 8:00	-0.393	0.015*	-0.159	0.341	-0.195	0.241	0.042	0.804	0.092	0.587	0.172	0.316	0.123	0.467
OCN	0.131	0.432	-0.012	0.942	0.298	0.069	0.240	0.147	-0.323	0.051	-0.250	0.141	-0.345	0.037*
Model 2														
Cortisol at 8:00	-0.391	0.020*	-0.136	0.436	-0.123	0.480	0.110	0.530	0.047	0.791	0.115	0.523	0.084	0.636
OCN	0.114	0.513	-0.046	0.795	0.308	0.072*	0.244	0.157	-0.334	0.053	-0.263	0.139	-0.326	0.060
Model 3														
Cortisol at 8:00	-0.389	0.041*	-0.072	0.716	-0.129	0.512	0.144	0.466	0.044	0.827	0.127	0.538	0.058	0.773
OCN	0.102	0.606	0.00	0.999	0.370	0.052	0.248	0.203	-0.426	0.027*	-0.363	0.069	-0.337	0.086

Model 1: bivariate correlation.

Model 2: controlling for age, sex, disease duration.

Model 3: controlling for age, sex, disease duration, PTH, calcium, phosphorus, and 25-hydroxyvitamin D [25(OH)D].

*p < 0.05.

SF, social function; RE, role of emotion; MH, mental health; VI, vitality; STAI-S/T, State–Trait Anxiety Inventory-State/Trait; BDI, Beck Depression Inventory; PHPT, primary hyperparathyroidism.

### OCN and Cortisol Levels Were the Determinants of Psychological Performance in Patients With Primary Hyperparathyroidism

As for the linear regression model of the BDI score, statistically significant variables were serum OCN (β = -0.422, p = 0.019) and cortisol at 0:00 (β = 0.371, p = 0.037), while variables excluded by collinearity analysis included age, serum PTH, 25(OH)D, calcium, cortisol at 8:00, and urinary free cortisol in 24 h. No significant variable was included in the equations of other questionnaires.

### Serum Cortisol and Osteocalcin Were Positively Correlated With Serum Calcium and Parathyroid Hormone Concentration in Primary Hyperparathyroidism Patients

In these 38 patients, we found that OCN was associated with PTH (r = 0.351, p = 0.031) and marginally associated with calcium (r = 0.304, p = 0.067), while cortisol was not related with these two parameters. In order to further test whether these findings could be replicated in a larger sample size, we further explored the relationships among OCN, cortisol, PTH, and calcium in 192 PHPT patients.

The baseline characteristics of 192 PHPT patients in different PTH tertile groups were shown in **Table 2**. The mean age of the cohort was 52.7 ± 13.8 years, with 76.6% (147/192) females. In terms of bone biochemical markers, it was found that, as the serum PTH concentration increased, the serum concentration of calcium (p = 0.000), PINP (p = 0.000), and β-CTX (p = 0.028) increased and phosphorus decreased (p = 0.000) significantly. Also, with the increase of PTH concentration, the percentage of patients with upper tertile of OCN concentration increased (p = 0.000), while both the serum 25(OH)D concentration (p = 0.000) and BMD (L1~L4) level (p = 0.000) declined significantly. As for cortisol, the serum cortisol at 0:00 (p = 0.021) was significantly elevated with the increase of PTH concentration. As serum PTH concentration increased, no difference in serum ACTH, cortisol concentration at 8:00 and 16:00, and urinary cortisol excretion in 24 h was observed (p > 0.05).

**Table 2:** Baseline characteristics of PHPT patients in different PTH tertile groups.

	Total	Tertile 1 (pg/ml)	Tertile 2 (pg/ml)	Tertile 3 (pg/ml)	p-value
		<167.50	167.50~318.90	>318.90	
Sex (female/total, %)	147/192, 76.6	54/64, 84.4	48/64, 75	45/64, 71.9	–
Age (years old)	52.7 ± 13.8	54.9 ± 12.2	51.2 ± 13.5	52.1 ± 15.5	0.286
Disease duration (months)	12 (0.25, 360)	16.5 (0.5, 240)	13.0 (0.25, 360)	12 (0.25, 360)	0.592
Weight (kg)	61.22 ± 10.81	61.57 ± 10.26	61.86 ± 11.34	60.29 ± 10.96	0.702
Height (cm)	162.56 ± 7.45	162.43 ± 6064	162.36 ± 7.68	162.89 ± 8.10	0.916
BMI (kg/m^2^)	23.01 ± 3.06	23.15 ± 3.07	23.43 ± 3.14	22.41 ± 2.92	0.213
SBP (mmHg)	128.52 ± 16.98	129.77 ± 20.52	126.84 ± 14.84	128.91 ± 15.40	0.613
HR (bpm)	75.12 ± 11.75	75.41 ± 9.92	74.20 ± 12.03	75.73 ± 13.27	0.760
Serum 25(OH)D (nmol/L)	35.13 ± 16.02	42.22 ± 16.07	33.67 ± 15.42	24.49 ± 14.02	0.000***
Serum PINP (ng/ml)	84.59 (17.96, 1,200)	69.11 (20.54, 207.90)	80.59 (17.96, 262.30)	151.80 (28.42, 1,200.0)	0.000***
Serum β-CTX (ng/ml)	0.84 (0.09, 4.06)	0.72 (0.28, 2.11)	1.01 (0.09, 2.80)	0.945 (0.14, 4.06)	0.028*
Serum OCN (upper tertile/total, %)	63/192, 32.8	7/64, 10.9	17/64, 26.6	39/64, 60.9	0.000***
Serum calcium(mmol/L)	2.74 ± 0.25	2.60 ± 0.17	2.72 ± 0.22	2.89 ± 0.27	0.000***
Serum phosphorus (mmol/L)	0.85 (0.44, 10.50)	0.97 (0.59, 1.27)	0.85 (0.54, 10.50)	0.76 (0.44, 1.20)	0.000***
BMD (L1~L4) (g/cm^2^)	0.957 ± 0.189	1.012 ± 0.162	0.992 ± 0.197	0.863 ± 0.173	0.000***
Albumin (g/L)	39.61 ± 3.58	39.78 ± 3.30	39.74 ± 3.60	39.31 ± 3.83	0.719
Serum creatinine(μmol/L)	65.52 ± 22.79	63.52 ± 16.48	65.57 ± 18.64	68.45 ± 30.59	0.445
Serum hemoglobin (g/L)	127.57 ± 20.14	129.23 ± 16.91	128.51 ± 21.15	125.03 ± 22.03	0.462
HbA1c (%)	5.55 ± 1.09	5.58 ± 0.65	5.39 ± 0.66	5.68 ± 1.66	0.338
Serum cortisol 8:00 (μg/dl)	11.28 ± 4.09	10.74 ± 3.32	11.05 ± 3.68	12.03 ± 5.02	0.175
Serum cortisol 16:00 (μg/dl)	5.28 (1.77, 21.19)	5.11 (2.19, 19.21)	5.02 (2.00, 15.76)	5.40 (1.77, 21.19)	0.859
Serum cortisol 0:00 (μg/dl)	2.65 (0.54, 18.84)	2.47 (0.54, 14.85)	2.46 (0.76, 18.12)	3.34 (0.96, 18.84)	0.021*
Urine cortisol (μg/24 h)	71.34 (1.90, 407.55)	70.00 (28.0, 144.96)	71.03 (3.31, 269.83)	73.16 (1.90, 407.55)	0.797
Serum ACTH (pg/ml)	25.83 (4.41, 105.92)	25.36 (4.41, 70.49)	26.20 (0.92, 105.92)	25.62 (6.4, 91.67)	0.681

BMI, body mass index; SBP, systolic blood pressure; HR, heart rate; 25(OH)D, 25-hydroxyvitamin D; PINP, Type I procollagen amino-terminal peptide; CTX-β, collagen I telopeptide-β;OCN, osteocalcin; BMD (L1~L4), bone mineral density (lumbar 1~4); HbA1c, glycosylated hemoglobin; ACTH, adrenocorticotropic hormone; PHPT, primary hyperparathyroidism.

*p < 0.05, ***p < 0.0001.

When patients were grouped according to tertiles of serum calcium concentration, as shown in **Table 3**, with the increase of serum calcium concentration, serum PTH (p = 0.000), PINP (p = 0.000), β-CTX (p = 0.001), and the percentage of patients with upper tertile of OCN concentration (p = 0.000) increased significantly, while serum phosphorus concentration (p = 0.002) and BMD (L1~L4) (p = 0.000) decreased significantly. Serum concentration of 25(OH)D was significantly (p = 0.000) different between calcium tertile groups. However, the differences in body weight, height, and BMI were not statistically significant. It was again shown that with the increase of calcium concentration, serum cortisol concentration at 8:00, 16:00, and 0:00 and urinary cortisol excretion did not show a statistically significant difference (p > 0.05).

**Table 3:** Baseline characteristics of PHPT patients in different calcium tertile groups.

	Total	Tertile 1 (mmol/L)	Tertile 2 (mmol/L)	Tertile 3 (mmol/L)	p-value
		<2.62	2.62~2.81	>2.81	
Sex (female/total, %)	147/192, 76.6	52/63, 82.5	47/66, 71.2	45/63, 71.4	–
Age (years old)	52.7 ± 13.8	54.9 ± 12.2	51.2 ± 13.5	52.1 ± 15.5	0.286
Disease duration (months)	12.0 (0.25, 360)	12.0 (0.25, 240)	12.0 (0.33, 180)	12.0 (0.25, 360.0)	0.986
Weight (kg)	61.22 ± 10.81	61.57 ± 10.26	61.86 ± 11.34	60.29 ± 10.96	0.702
Height (cm)	162.56 ± 7.45	162.43 ± 6.64	162.36 ± 7.68	162.89 ± 8.10	0.916
BMI (kg/m^2^)	23.01 ± 3.06	23.15 ± 3.07	23.43 ± 3.14	22.41 ± 2.92	0.213
SBP (mmHg)	128.52 ± 16.98	129.77 ± 20.25	126.84 ± 14.84	128.91 ± 15.40	0.613
HR (bpm)	75.12 ± 11.75	75.41 ± 9.92	74.20 ± 12.03	75.73 ± 13.27	0.760
Serum PTH (pg/ml)	220.35 (74.70, 218.10)	149.5 (74.7, 1,939.9)	215.65 (94.30, 2,380.10)	398.2 (88.9, 2,680.1)	0.000***
Serum 25(OH)D (nmol/L)	35.13 ± 16.02	42.22 ± 16.07	33.67 ± 15.42	29.49 ± 14.02	0.000***
Serum PINP (ng/ml)	84.59 (17.96, 1,200)	69.11 (17.96, 418.90)	86.92 (24.09, 479.20)	110.7 (28.95, 1200)	0.000***
Serum β-CTX (ng/ml)	0.835 (0.09, 4.06)	0.703 (0.11, 1.91)	0.93 (0.24, 1.95)	1.44 (0.09, 4.06)	0.001**
Serum OCN (upper tertile/total,%)	63/192, 32.8	11/63, 17.5	20/66, 30.3	32/63, 50.8	0.000***
Serum phosphorus (mmol/L)	0.85 (0.44, 10.50)	0.94 (0.44, 10.50)	0.85 (0.44, 1.32)	0.79 (0.50, 1.20)	0.002**
BMD (L1~L4) (g/cm^2^)	0.957 ± 0.189	1.012 ± 0.162	0.992 ± 0.197	0.863 ± 0.1730	0.000***
Albumin (g/L)	39.61 ± 3.58	39.78 ± 3.30	39.74 ± 3.60	39.31 ± 3.83	0.719
Serum creatinine (μmol/L)	65.52 ± 22.79	63.52 ± 16.48	64.57 ± 18.64	68.45 ± 30.59	0.445
Serum hemoglobin (g/L)	127.57 ± 20.14	129.23 ± 16.91	128.51 ± 21.15	125.03 ± 22.03	0.462
HbA1c (%)	5.55 ± 1.09	5.58 ± 0.65	5.39 ± 0.66	5.68 ± 1.66	0.338
Serum cortisol 8:00 (μg/dl)	11.28 ± 4.09	10.74 ± 3.32	11.05 ± 3.68	12.03 ± 5.02	0.175
Serum cortisol 16:00 (μg/dl)	5.28 (1.77, 21.19)	4.71 (2.19, 19.21)	5.67 (2.00, 12.72)	5.56 (1.77, 21.19)	0.133
Serum cortisol 0:00 (μg/dl)	2.65 (0.54, 18.84)	2.61 (0.54, 14.85)	2.46 (0.91, 12.35)	2.91 (0.99, 18.84)	0.092
Urine cortisol (μg/24h)	71.34 (1.90, 407.55)	71.78 (43.44, 157.92)	71.72 (1.90, 222.14)	70.92 (3.31, 407.55)	0.999
Serum ACTH (pg/ml)	25.83 (4.41, 105.92)	25.48 (7.06, 91.67)	26.23 (4.41, 72.58)	23.82 (10.4, 105.92)	0.419

BMI, body mass index; SBP, systolic blood pressure; HR, heart rate; 25(OH)D, 25-hydroxyvitamin D; PINP, Type I procollagen amino-terminal peptide; CTX-β, collagen I telopeptide-β: OCN, osteocalcin; BMD (L1~L4), bone mineral density (lumbar 1~4); HbA1c, glycosylated hemoglobin; ACTH, adrenocorticotropic hormone; PHPT, primary hyperparathyroidism.

**p < 0.01, ***p < 0.0001.

In addition, in the bivariate correlation model between serum PTH concentration and OCN, cortisol concentration showed that serum PTH was positively and significantly correlated with serum OCN (r = 0.373, p = 0.000). When controlling for age, sex, and disease duration, and additionally with phosphorus, calcium, and 25(OH)D, PTH was still positively and significantly correlated with serum OCN concentration (r = 0.323, p = 0.000). However, serum PTH concentration was not significantly correlated with serum cortisol of different time points and urinary cortisol excretion (p > 0.05) in the bivariate and partial correlation model.

As to serum calcium level, it was positively and significantly correlated with serum OCN (r = 0.240, p = 0.000) and cortisol concentration at 0:00 (r = 0.246, p = 0.001). When controlling for age, sex, and duration, the correlation of serum calcium with OCN (r = 0.248, p = 0.001) and cortisol at 0:00 (r = 0.249, p = 0.001) was still significantly positive. When serum 25(OH)D and phosphorus were adjusted, serum calcium was still significantly and positively correlated with serum cortisol at 0:00 (r = 0.251, p = 0.001) and OCN (r = 0.222, p = 0.003). Even when serum PTH was further adjusted, the correlation between serum calcium and cortisol at 0:00 was still significantly positive (r = 0.245, p = 0.001), while the correlation between serum calcium and OCN (r = 0.110, p = 0.143) lost its significance.

## Discussion

The findings from this study lie in two aspects. Firstly, the balance of serum OCN and cortisol was associated with the psychological performance in PHPT patients. Secondly, with the elevation of serum calcium and PTH, the concentration of serum cortisol and OCN increased.

The effects of glucocorticoid (15–17, 19) and OCN (21, 23, 28) on the depression and anxiety symptoms in patients and related behavioral performance in animals were widely reported. Thus, changes of cortisol and OCN in PHPT patients and their relationships with scores of questionnaires were investigated in this study. We found that as serum PTH concentration increased, circulating OCN and cortisol levels increased significantly. Besides, through the correlation analysis, we found that cortisol and OCN were negatively and positively related with psychological performance in PHPT patients, respectively, independent of PTH and calcium.

To the best of our knowledge, the balance of cortisol and OCN has not been reported in studies concerning psychological performance in PHPT so far. In our study, we found that OCN and cortisol were two determinants of the psychological performance in PHPT patients, independent of PTH and calcium. Our finding was supported by previous clinical and animal studies. First of all, clinical studies revealed the opposite effects of cortisol and OCN on psychological performance and brain structure that was related to affective disorders. On the one hand, excessive cortisol was related to decreased volume of different brain areas of patients. It was reported that hypercortisolemia in CS leads to shrinkage of amygdala volume (29), an important brain structure involved in emotional response (30) and a target of cortisol hormone with abundant glucocorticoid receptors (31). It was also reported that amygdala volume was negatively and significantly correlated with scores of STAI-S and BDI in CS (29). Other findings in active CS patients revealed smaller volumes in comparison to healthy controls in gray matter of the medial frontal gyrus (32), cerebellar cortex, and gray matter volumes (33), which was observed in depression models (34). Besides, higher serum cortisol at bedtime could reflect a flatter diurnal slope (35, 36), which was associated with an impaired psychological manifestation (37). In this study, we found that a flatter diurnal slope reflected by higher serum cortisol concentration at 0:00 was associated with worse psychological performance in PHPT patients. On the other hand, a recent study (38) revealed that OCN concentration was lower in depressive patients than that in healthy controls. In obese and control human subjects, lower serum levels of OCN were associated with lower cognitive performance together with cognitive and depressive brain microstructural changes, and serum OCN independently explained 10% of the variation in cognitive performance (28). The correlation between the decrease of OCN concentration and cognition impairment has also been noted in older adults (39). Secondly, experimental studies uncovered molecular mechanisms of harmful and protective effects of glucocorticoid and OCN for psychological features, respectively. Glucocorticoid was reported to be increased in depression and anxiety mice and was revealed to increase the hippocampus apoptosis *in vivo* and *in vitro* (40). In contrast, OCN was demonstrated to exhibit neuron-protective effects on dopaminergic neuron in a Parkinson’s disease mouse model through regulating gut microbiota in our most recent study (41). It was also reported (40) that glucocorticoid elevation leads to decreased expression of brain-derived neurotrophic factor (BDNF) in depressive mouse hippocampus and in PC12 cells, while OCN exerted a protective effect for depression and anxiety by increasing BDNF expression through activation of cAMP/PKA signaling in mice (21). Thus, the above studies suggested that opposite to glucocorticoid, OCN might exert protective effects on depression and anxiety in patients and mice. The net effect of this balance may explain the inconsistent findings regarding the psychological performance in PHPT from different studies (7–9). Also, since there is no report on changes of these parameters before and after surgery in PHPT patients, it is thus interesting to investigate whether its dynamic changes are related to the psychological performance in PHPT patients after parathyroidectomy (1, 9, 10).

It is noteworthy that in our multivariate correlation analysis, serum calcium rather than PTH was independently and positively correlated with cortisol concentration in PHPT patients. From a clinical perspective, Espiritu et al. (41) reported that PHPT patients with serum calcium over 2.47 mmol/L had more symptoms of depression than patients with lower calcium. Weber et al. (42) also found that serum calcium rather than PTH was related to depression. These observations were in line with our results that higher calcium was independently related to higher serum cortisol level, which was associated with more depression symptoms, as evidenced by SF score. From the perspective of mouse studies, serum calcium can stimulate the secretion of adrenal hormones, while PTH just acts like a calcium ionophore (43). To some extent, this finding could partly explain why no difference in depression and cognitive indices was reported in mild hypercalcemic and normocalcemic PHPT patients (7).

However, Bargren et al. (44) found that patients with milder hypercalcemia had more depression, suggesting that hypercalcemia might not mediate these symptoms. In our study, it was demonstrated that with the elevation of serum calcium level, both serum cortisol and OCN increased, thus it is of interest to investigate whether the findings from Bargren et al. (44) could be explained by the balance of cortisol and OCN. In addition, in the other study of Kearns et al. (11), baseline PTH level, but not calcium, was found to have a weak relationship with change in depressive symptoms after parathyroid surgery. This result seemed to contradict with our findings. In our study, both OCN and cortisol were increased along with the increase of PTH; however, after controlling PTH, although serum calcium was not related to OCN, it still significantly correlated with midnight serum cortisol level, while morning cortisol level was in a significantly negative association with SF independent of PTH. Furthermore, in our study, neither PTH nor calcium has any correlation with scores in questionnaires. Thus, investigating the relative contribution of PTH, calcium, OCN, and cortisol to the development of anxiety and depression behaviors in PHPT in a larger cohort, especially before and after parathyroidectomy, is very important.

Although we revealed the presence of psychoneurological phenotypes in PHPT and found the independent role of serum cortisol and OCN in PHPT patients, this study was just exploratory or hypothesis generating, instead of a confirmatory investigation. Some limitations should be mentioned here. First, the postoperative concentrations of cortisol and OCN as well as the psychological questionnaires were not examined and compared with the preoperative ones. Thus, we have no idea whether the balance of these two markers and patients’ psychological scores changed or not after parathyroidectomy. Second, in this study, we analyzed concentrations of total OCN, rather than uncarboxylated OCN (ucOCN), which is a metabolically active form of OCN at least in mouse studies (21). Third, the sample size of this study, especially those receiving questionnaires, was small; selection bias should be considered. In our sensitivity analysis, it was found that no significant difference of serum OCN and cortisol existed between those receiving questionnaires and their counterparts. Last, we did not measure body water distribution in PHPT patients who were usually treated with water repletion. It was recently shown that the ratio of extracellular water to total body water was related to cognitive function in diabetes patients (45).

To sum up, in this study, it was demonstrated that the serum levels of OCN and cortisol were independently associated with the development of psychological symptoms in PHPT patients. More basic and clinical studies are needed to test and verify this observation.

## Data Availability Statement

The raw data supporting the conclusions of this article will be made available by the authors, without undue reservation.

## Ethics Statement

The studies involving human participants were reviewed and approved by Shanghai Ruijin Hospital. Written informed consent for participation was not required for this study in accordance with the national legislation and the institutional requirements.

## Author Contributions

J-ML and L-HS conceived the project. S-MW carried out most of the information collection and data analysis and wrote the manuscript. YH and M-TZ collected part of patients’ clinical information. BT and H-YZ played a key role in maintaining and screening patients. All authors contributed to the article and approved the submitted version.

## Funding

This study was funded by the Yang Fan Project of Shanghai Science and Technology Commission (19YF1430000).

## Conflict of Interest

The authors declare that the research was conducted in the absence of any commercial or financial relationships that could be construed as a potential conflict of interest.

## Publisher’s Note

All claims expressed in this article are solely those of the authors and do not necessarily represent those of their affiliated organizations, or those of the publisher, the editors and the reviewers. Any product that may be evaluated in this article, or claim that may be made by its manufacturer, is not guaranteed or endorsed by the publisher.
