# Supplementary material for: The Associations of Serum Osteocalcin and Cortisol Levels With the Psychological Performance in Primary Hyperparathyroidism Patients
Source: Front Endocrinol (Lausanne). 2021 Aug 12;12:692722. doi: 10.3389/fendo.2021.692722 (PMC8397408; doi:10.3389/fendo.2021.692722)
Supplement: Supplementary file 1 [file Table_1.docx]

Supplement table. Comparison between baseline characteristics of PHPT patients receiving and not receiving questionnaires

|  | Receiving | Not receiving | P value |
| --- | --- | --- | --- |
| Sex (female/total,%) | 30/38, 78.9 | 12/14, 85.7 | - |
| Age (years old) | 56.97±13.41 | 54.07±12.38 | 0.483 |
| Duration (months) | 10(0.25,360) | 22 (1.50,96) | 0.613 |
| Weight (kg) | 60.06±10.07 | 59.93±8.43 | 0.969 |
| Height (cm) | 162.25±8.57 | 163.33±5.33 | 0.605 |
| BMI (kg/m^2^) | 22.69±2.98 | 22.56±2.92 | 0.889 |
| SBP (mmHg) | 134.76±18.31 | 132.50±17.81 | 0.692 |
| HR (bpm) | 76.82±10.12 | 77.79±9.12 | 0.755 |
| PTH (pg/ml) | 235.6(94.6,1304.7) | 167.8(99.7,513.4) | 0.092 |
| Serum 25-(OH)D (nmol/L) | 42.22±16.31 | 37.69±15.92 | 0.380 |
| Serum β-CTX (ng/ml) | 1.03±0.59 | 0.69±0.34 | 0.130 |
| Serum OCN (upper tertile/total,%) | 14/38, 36.8 | 2/14, 14.3 | 0.323 |
| Serum calcium(mmol/L) | 2.77±0.23 | 2.56±0.12 | 0.006** |
| Serum phosphorus (mmol/L) | 0.85±0.20 | 0.91±0.17 | 0.406 |
| BMD (L1~4) (g/cm^2^) | 0.92±0.19 | 1.01±0.17 | 0.150 |
| Albumin (g/ L) | 40.66±4.13 | 39.50±3.06 | 0.345 |
| Serum creatinine(umol/L) | 71.13±32.76 | 62.50±3.06 | 0.359 |
| Serum hemoglobin (g/ L) | 126.19±16.63 | 129.08±11.86 | 0.568 |
| HbA1c(%) | 5.63±1.43 | 5.60±1.27 | 0.934 |
| Serum cortisol 8:00(ug/dl) | 12.14±4.20 | 12.20±2.70 | 0.959 |
| Serum cortisol 16:00(ug/dl) | 6.18±2.38 | 4.85±1.64 | 0.269 |
| Serum cortisol 0:00 (ug/dl) | 2.45(0.99,18.84) | 1.86(0.96,8.30) | 0.261 |
| Urine cortisol(ug/24h) | 69.27(24.46,304.46) | 68.16(45.54,99.59) | 0.413 |
| Serum ACTH (pg/ml) | 21.48(4.41,79.43) | 25.33(16.40,52.65) | 0.210 |

P<0.01 ** P<0.0001

BMI: body mass index; SBP: systolic blood pressure; HR: heart rate; 25-(OH)D:25-hydroxyvitamin D; β-CTX: Collagen I telopeptide-β；OCN：osteocalcin; BMD(L1~4): bone mineral density (lumbar1~4); HbA1c: glycosylated Hemoglobin; ACTH: adrenocorticotropic hormone
